# Evaluation of Two Methods to Estimate and Monitor Bird Populations

**DOI:** 10.1371/journal.pone.0003047

**Published:** 2008-08-27

**Authors:** Sandra L. Taylor, Katherine S. Pollard

**Affiliations:** Department of Statistics, University of California Davis, Davis, California, United States of America; University of Sheffield, United Kingdom

## Abstract

**Background:**

Effective management depends upon accurately estimating trends in abundance of bird populations over time, and in some cases estimating abundance. Two population estimation methods, double observer (DO) and double sampling (DS), have been advocated for avian population studies and the relative merits and short-comings of these methods remain an area of debate.

**Methodology/Principal Findings:**

We used simulations to evaluate the performances of these two population estimation methods under a range of realistic scenarios. For three hypothetical populations with different levels of clustering, we generated DO and DS population size estimates for a range of detection probabilities and survey proportions. Population estimates for both methods were centered on the true population size for all levels of population clustering and survey proportions when detection probabilities were greater than 20%. The DO method underestimated the population at detection probabilities less than 30% whereas the DS method remained essentially unbiased. The coverage probability of 95% confidence intervals for population estimates was slightly less than the nominal level for the DS method but was substantially below the nominal level for the DO method at high detection probabilities. Differences in observer detection probabilities did not affect the accuracy and precision of population estimates of the DO method. Population estimates for the DS method remained unbiased as the proportion of units intensively surveyed changed, but the variance of the estimates decreased with increasing proportion intensively surveyed.

**Conclusions/Significance:**

The DO and DS methods can be applied in many different settings and our evaluations provide important information on the performance of these two methods that can assist researchers in selecting the method most appropriate for their particular needs.

## Introduction

Estimating and monitoring bird populations are important components of conservation and management programs focused on birds as well as for broader ecosystem-focused programs. In these programs, effective management depends upon accurately estimating trends in abundance over time, and in some cases estimating abundance. Survey methods that yield relative abundance indices (e.g., point counts) commonly are used to assess the status of populations and to track temporal changes in abundance. However, an index can accurately reflect population trend only if the expected value of the ratio of the index to the true population does not change over time [1]. When the detection probability varies among surveys, the ratio of the index to the true population varies. As a result, trends in the index over time can reflect variation in detection probability rather than true trends in abundance. This situation can lead to incorrect conclusions regarding a population's size and trajectory, possibly resulting in the implementation of inappropriate management actions. Thus, use of indices for monitoring population trends of birds and other wildlife has been criticized [2]–[5]. To overcome the short-comings of index methods, several authors recommend using methods that include estimation of detection probabilities and result in direct estimates of abundance [4], [6]–[9].

Traditional methods for estimating abundance include capture-recapture [10]–[12], removal [13] and distance-sampling methods [14]. Capture-recapture and removal methods can be infeasible or cost prohibitive for avian monitoring programs. Distance sampling methods require an accurate estimate of the distance between the observer and each bird. Accurate distance estimates can be challenging to obtain for some species or environments and can differ considerably between observers.

Here we consider, two methods that have been proposed for avian monitoring programs: the double sampling (DS) method [5] and double observer (DO) method [2]. These methods are less time and cost intensive than capture-recapture and removal methods, and unlike distance sampling methods, they do not require a distance estimate. Both methods have been used to estimate density and population size in general avian studies as well as in species-specific studies in a range of habitats [2], [15]–[19]. The successful application of these methods to a range of species in a variety of habitats indicates that both methods are logistically feasible and cost-effective in many situations. However, the relative merits and short-comings of the two methods continue to be the subject of debate. An understanding of the precision and accuracy of these two methods under various conditions is important additional information to consider when choosing an estimation method. Our goal is to offer an unbiased assessment of the performance of the DS and DO methods through computer simulations.

In the DS method, two survey techniques are used: (1) a rapid, inexpensive technique and (2) a more intensive and expensive technique. The rapid method is applied over a large area while the intensive method is used on a subset of the areas surveyed with the rapid method. Detection probability is estimated from the units surveyed with both rapid and intensive methods and the estimate of detection probability is used to convert the counts from the rapid method to a population estimate. This method produces unbiased population estimates if density estimates from the units surveyed with the intensive method are unbiased and the rapid and intensive survey units are randomly selected [9].

In the DO method, two observers, a primary and a secondary observer, survey an area together [2]. The primary observer identifies the number and species of all birds that are observed and communicates this information to the secondary observer. The secondary observer also surveys the area but does not communicate detections to the primary observer. The secondary observer records the primary observer's detections and any birds the secondary observer detected but the primary did not. The observers switch roles such that each serves as the primary for about half of the survey. Detection probabilities are estimated for each observer based on the number of birds seen by the secondary observer that are not seen by the primary observer. The overall detection probability is calculated from the observer-specific detection probabilities as the probability that a bird is detected by at least one observer. Population size is estimated from the detection probability and total number of birds observed. It is important to note that the DO method estimates the size of the population that is *observable* at the time of the survey, sometimes referred to as the conspicuous population. When some portion of the population is not observable during the survey, estimates generated with the DO method do not reflect the entire population.

We used computer simulations to compare population estimates based on DO and DS methods for a range of detection probabilities, survey proportions, and spatial distributions. In addition, for the DO method we investigated the effect of different detection probabilities for each observer. For the DS method we consider the influence of the proportion intensively surveyed on the precision and accuracy of the population estimates. Computer simulations provide an unbiased means to assess and compare the performance of the DO and DS methods across a range of realistic scenarios. Simulations are an ideal method for evaluating the performance of alternative estimators because the methods can be applied to populations of known size. The bias and variance of estimates from different methods then can be determined and compared. Further, the sensitivity of the estimators to variations in detection probability, proportion of area surveyed, and the spatial distribution of the population within the survey area can be experimentally evaluated.

## Results

We used simulations to compare the performance of the DO and DS methods under a range of realistic survey scenarios. The results did not differ materially between simulations in which we assumed all birds to have the same detection probability and those in which the detection probability varied randomly among individuals. Thus, results are presented only for the simulations with constant detection probability.

### Overall comparison of population estimates

We compared the DS and DO methods in terms of bias, variability, and percentage of 95% confidence intervals that encompass the true population size. The DS method provided unbiased population estimates regardless of variation in population clustering, detection probabilities and survey proportions. Population estimates for the DO method also were centered on the true population size, except when detection probabilities were below 30% (see below). Variability of population estimates across simulation runs was similar for both methods, except at lower detection probabilities (<50%), where the DS method was less variable. Neither method provided 95% confidence intervals with the expected 95% coverage. Whereas coverage probabilities were only slightly low for the DS method, the DO method had highly variable confidence interval coverage and often the confidence intervals did not encompass the true population size. The impacts of detection probability, population clustering, and survey proportion on these overall patterns are discussed in the following sections.

### Detection probability

**Figure 1:**
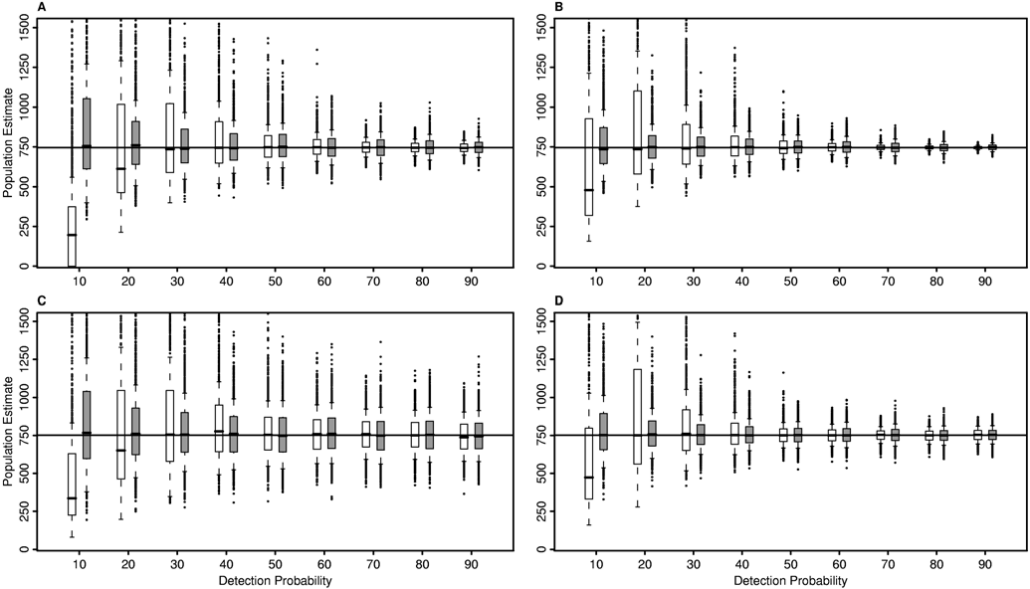
Population estimates for varying survey proportions. Population estimates were obtained based on the double observer and double sampling methods for 1,000 simulations each for the low clustered population with survey proportions of 25% and 75% (Panels A and B, respectively) and for the high clustered population with survey proportions of 25% and 75% (Panels C and D, respectively). White indicates the double observer method; gray indicates the double sampling method. Horizontal lines show true population sizes. The double observer method generally produced biased estimates when the detection probability was below 30%.

**Figure 2:**
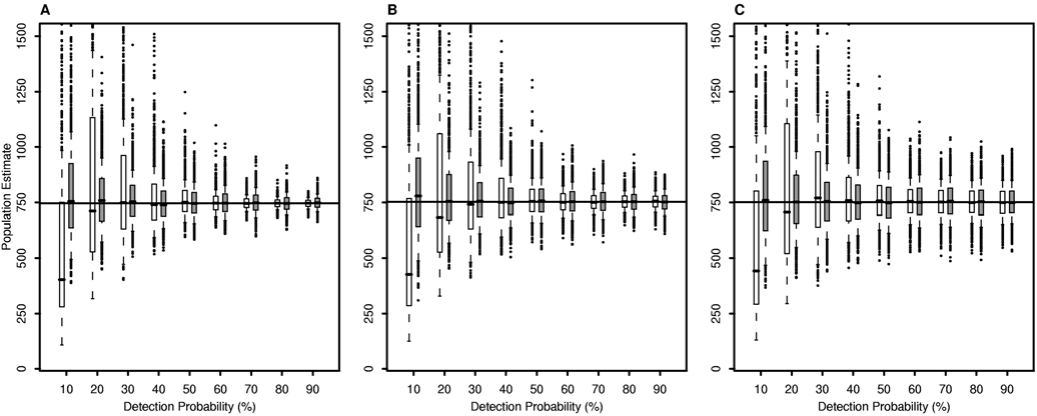
Population estimates for varying levels of population clustering. Population size was estimated using the double sampling and double observer methods for 1,000 simulations each for the low, moderate, and high clustered populations (Panels A, B, and C, respectively) with the survey proportion at 50%. White indicates the double observer method; gray indicates the double sampling method. Horizontal lines show true population sizes. The double observer method yielded more variable population estimates than the double sampling method for detection probabilities below 50% regardless of population clustering.

Of the variables we investigated, detection probability had the largest impact on population estimates. In terms of bias, detection probabilities did not affect the DS method. The DO method usually underestimated the population when detection probability was 10 or 20% (Figures 1 and 2). For detection probabilities greater than 20%, the median bias was small for both methods, ranging from −0.9 to 1.3% for the DS method and from −5.7 to 3.5% for the DO method. In contrast, at a detection probability of 10%, the DO method underestimated the population with a median bias ranging from −57 to −36% whereas the DS method remained unbiased.

**Figure 3:**
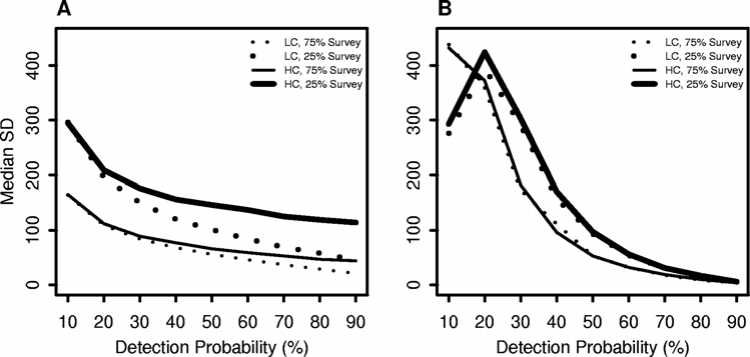
Median standard deviation of population estimates. The figures show the changes in median standard deviation (SD) relative to detection probability for the low (LC) and high clustered (HC) populations at survey proportions of 25% and 75% for the double observer and double sampling methods (Panels A and B, respectively). Median standard deviation for the double observer method is larger than for the double sampling method at low detection probabilities but is very small at high detection probabilities.

For both methods, variability in population estimates among runs decreased as detection probability increased (Figures 1 and 2). This reduction was most pronounced for the DO method. At high detection probabilities, DO population estimates were in a narrow range and the median SD across runs was small regardless of population clustering and survey proportion (Figure 3). Population estimates were much more variable and some estimates of the DO method were extremely large at detection probabilities less than 40%. The DS method also yielded some large population estimates for low detection probabilities, but the estimates were not as extreme as the DO method estimates. The median bias of population estimates from the DS method declined with detection probability but to a smaller degree than the DO method (Figure 3). Thus, estimates from the DS method were less variable than the DO method at low detection probabilities but the DO method yielded less variable estimates than the DS method at high detection probabilities.

**Figure 4:**
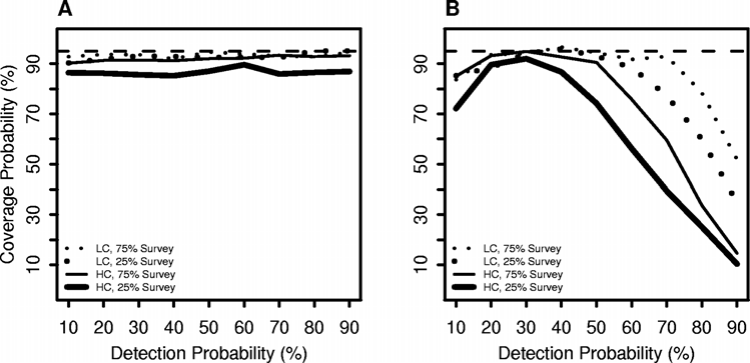
Changes in coverage probabilities of 95% confidence intervals for population estimates. The figures show the changes in coverage probability relative to detection probability for the low (LC) and high clustered (HC) populations at survey proportions of 25% and 75% for the double observer and double sampling methods (Panels A and B, respectively). The dashed line indicates 95% level. Coverage probability for the double sampling method remains close to the nominal level for all detection probabilities and populations but declines to less than 50% for the double observer methods at high detection probabilities.

In terms of confidence interval coverage, the DS and DO methods showed different patterns across the range of detection probabilities. For the DS method, increasing detection probability resulted in better coverage, although differences in coverage among detection probabilities were small (less than 5%) for a given survey proportion and population clustering level (Figure 4). For the DO method, coverage probabilities were closest to the nominal level for detection probabilities between 30 and 50% but declined substantially for higher and lower detection probabilities (Figure 4).

### Population clustering

The variability of population estimates increased as the level of clustering in the population increased for both methods (Figures 1 and 2). For example, for the HC population of 751 birds, with 25% of the area surveyed and a detection probability of 70%, DO population estimates ranged from 411 to 1,143 birds, whereas for the corresponding LC population the estimates ranged from 622 to 918 birds. The DS method showed a similar pattern and degree of variability. Above a detection probability of 50%, the methods resulted in similar distributions of population estimates for the HC and medium clustered (MC) populations, but the DO method yielded slightly less variable population estimates than the DS method for the LC population. For both methods, confidence interval coverage decreased with the level of population clustering (Figure 4).

### Proportion of population surveyed

Increasing the survey proportion from 25 to 75% generally resulted in less variable population estimates and better confidence interval coverage with both methods (Figure 4). We observed a relatively large reduction in the median SD of population estimates at all detection probabilities for the DS method with increases in survey proportion (Figure 3). However, for the DO method the effect of increasing the survey proportion was most evident at intermediate detection probabilities.

### Observers with different detection probabilities (DO method only)

Differences in detection probabilities between the two observers did not substantially affect population estimates of the DO method. The overall probability that a bird will be detected by one of the observers is 1−((1−*p*_1_)(1−*p*_2_)) where *p*_1_ and *p*_2_ are the detection probabilities for observer 1 and 2, respectively. For similar overall detection probabilities, population estimates were similar when observers had equal detection probabilities to when detection probabilities differed by up to 60% (Table 1). Variance estimates tended to be larger when detection probabilities differed between the observers but the pattern was not consistent and differences were not large (Table 1).

**Table 1:** Median population estimates (SD) for the double observer method.

Observer Detection Probability	LC Population	HC Population
	Actual population = 746	Actual population = 751
Overall detection probability≈64%		
p_1_ = p_2_ = 40%^a^	753 (118)	750 (115)
p_1_ = 30%, p_2_ = 50%	744 (113)	760 (121)
p_1_ = 20%, p_2_ = 60%	741 (115)	755 (119)
Overall detection probability≈84%		
p_1_ = p_2_ = 60%	742 (38)	748 (38)
p_1_ = 40%, p_2_ = 70%	745 (48)	750 (49)
p_1_ = 30%, p_2_ = 80%	747 (45)	755 (45)
p_1_ = 20%, p_2_ = 80%	745 (61)	753 (60)

a p_1_ and p_2_ are the detection probabilities for observers 1 and 2, respectively.

The overall detection probability was near 64 or 84% with equal and or unequal detection probabilities for the two observers. Results are provided for the low (LC) and high (HC) clustered populations. Proportion surveyed was 50%. Values are based on 1,000 simulated surveys.

### Proportion of units surveyed (DS method only)

Population estimates were unbiased for the DS method when the proportion of units intensively surveyed was 30 or 50% (Table 2). With only 10% of the units intensively surveyed however, population estimates were biased high for low detection probabilities; this bias declined with increasing detection probability. This pattern occurred for all levels of population clustering. As the proportion of intensively surveyed units increased, the SD of the population estimate declined (Table 2). Increasing the proportion intensively surveyed from 10 to 30% resulted in up to a 50% decrease in the median SD.

**Table 2:** Median population estimates (SD) for the double sampling method.

Detection Probability	10% Intensive	30% Intensive	50% Intensive
	Estimate (SD)	Estimate (SD)	Estimate (SD)
Low Cluster Population (Actual population = 746)			
20%	751 (237)	739 (119)	747 (80)
40%	746 (141)	750 (75)	746 (51)
60%	740 (94)	744 (51)	750 (37)
80%	744 (60)	746 (35)	745 (27)
High Cluster Population (Actual population = 751)			
20%	766 (215)	745 (133)	753 (102)
40%	754 (138)	753 (98)	752 (84)
60%	747 (107)	749 (84)	750 (76)
80%	754 (81)	747 (73)	749 (72)

Values are for low (LC) and high clustered (HC) populations with different proportions intensively surveyed and detection probabilities. Proportion surveyed with the rapid method was 50%. Values are based on 1,000 simulated surveys.

## Discussion

### Double sampling method

A necessary condition for the DS method to provide an unbiased estimate of the total population is that the intensive method yields an unbiased estimate of the total number of birds in units surveyed. If some birds present in the survey unit are not detected with the intensive method, the population will be underestimated. The magnitude of the underestimation is directly related to the proportion of the birds detected with the intensive method. If only 90% of the birds in intensively surveyed units are observed, population estimates will on average be 90% of the actual population. Thus, for studies where population size is of primary importance, the DS method will provide reliable estimates only if the intensive method provides an unbiased estimate of all birds present in units surveyed. However, as long as the proportion of birds detected with the intensive method remains constant over time, the DS method will provide an unbiased estimate of population trend.

Population estimates remained essentially unbiased with changes in the detection probability. Even at the low detection probability of 10%, the DS method provided essentially unbiased estimates. Thus, as long as the intensive method yields an unbiased estimate, the DS will provide unbiased population estimates even when the rapid technique has a low probability of detection.

Population estimates did not change substantially as the proportion of the study area increased. However, the SD of these estimates declined with increases in the survey proportion resulting in more precise estimates. Similarly, as the proportion of the study area surveyed with the intensive method increased, the SD of the estimates declined. In contrast, we found that the SD increased with increased population clustering. Thus, for species with highly clustered distributions, increasing the proportion intensively surveyed and the proportion of the study area surveyed could help achieve an acceptable variance estimate.

In our simulations, confidence intervals for DS estimates were often a little below the 95% nominal coverage. We found that coverage probability was lowest when the survey proportion and detection probability was 25% and also for the HC population. Confidence interval coverage is determined by the bias, variance and the distribution of the estimate. Since the estimates were essentially unbiased, the low coverage probability could result from either underestimating the variance or deviations from normality. Population estimates deviated from normality when the survey proportion and detection probability were low but otherwise appeared substantially normally distributed. Thus, underestimating the variance of the population estimate appears to be the primary cause of the low coverage probability although deviations from normality likely contributed in some cases.

Cochran [20] suggested that the coefficients of variation for the mean number of birds observed with the intensive method (*y̅*) and the rapid method (*x̅*) needed to be less than 0.1 for the variance approximation to be appropriate. The coefficients of variation for both *y̅* and *x̅* generally exceeded 0.1 for the MC and HC populations in our study. This suggests that the low coverage probability for these populations indeed resulted from underestimating the variance. For the LC population, the coefficients of variation usually were less than 0.1 and coverage probability was closer to the nominal level. In real populations, birds tend to be highly clustered. Hence, the coverage probabilities of confidence intervals from field studies may typically be less than the nominal level.

With the DS method, a population estimate cannot be calculated if no birds are observed with the rapid method in units surveyed with both the rapid and intensive methods or if no birds are observed with the intensive method. In our simulations, these circumstances were most likely to happen with the HC population and a survey proportion of 25% because the HC population contained many survey units with no birds. In field studies, no detections of birds in intensively surveyed units also could happen if the species occurs at low densities throughout the study area. Species present at low densities present challenges for all estimation methods, but for highly clustered populations, a stratified sampling approach can minimize the potential of not observing any birds in the intensive units in the study.

### Double observer method

Population size estimates with the DO method were centered on the true population at detection probabilities greater than 20%. However, the variability of the population estimates differed substantially with detection probability for all levels of population clustering and survey proportions. At low detection probabilities (less than 40%), population estimates were highly variable with some large estimates (>20,000 birds). Nichols et al. [2] recommended using the DO method only when the detection probability exceeded 40%. In our study, population estimates based on the DO method were highly variable at detection probabilities below 40%. At a detection probability of 50%, the two methods showed similar variability in estimates among simulated surveys, suggesting that Nichols et al.'s threshold of 40% is appropriate.

The DO method estimates the observable component of the population which is the entire population in our simulations. In field studies, if a portion of the population is not observable, the DO method will underestimate the true population. This effect would be similar to the effect of not observing all birds with the intensive survey method under the DS method. Like the DS method, the DO method will yield unbiased estimates of the entire population only if all individuals have the potential to be observed with the survey techniques. If the proportion of the population that is observable does not vary among surveys, the DO method can be used to monitor trends.

An assumption of the DO method is that the probability of detection of all individuals of the same species is the same. In many field situations, this assumption likely does not hold. For example, individuals closer to the observer could be more likely to be detected than those farther away from the observer. When we allowed the detection probability to vary among individuals in our simulations, population estimates were similar to those obtained assuming constant inter-individual detection probability. This result indicates that the DO method can perform adequately in field situations where the detection probability varies among individuals.

The coverage probability of confidence intervals for the DO method was highly variable among the simulations. Counter intuitively, as the detection probability increased, the coverage probability declined. Coverage probability also varied with the level of population clustering and the survey proportion. The pattern in coverage probability results primarily from changes in the variance of estimates with the level of population clustering, detection probability and survey proportion. At high detection probabilities in particular, the variance of the population estimate was small resulting in narrow confidence limits. Because of the narrow confidence limits, over or underestimating the true population by even a small amount resulted in the confidence interval not encompassing the true population. The highest coverage probability occurred at detection probabilities of 30% to 50%. At this level, population estimates were essentially unbiased and the confidence limits were wide enough to compensate for deviations of estimates from the true value. At smallest the detection probabilities (10 and 20%), variance estimates were large, but the bias also was large.

Differences in detection probabilities between the two observers did not adversely affect the performance of the DO estimators. The precision and accuracy of the estimates were more strongly affected by the overall detection probability rather than the degree of difference between the two observers. These results suggest that when the detection capabilities of observers differ, better results will be obtained if strong observers are paired with poor observers rather than pairing similarly skilled observers. Further, training that improves the detection capabilities of all observers will improve the precision and accuracy of population estimates.

Nichols et al. [2] noted that if one observer does not detect any birds as either the primary or secondary observer the variance of the population estimate is undefined. It also is undefined if neither observer detects any novel individuals as the secondary observer. In the latter case, the primary observer counts provide the best estimate of the number of birds present in the units surveyed. Without a variance estimate, however, confidence limits for this estimate cannot be developed. We suggest a bootstrap approach to generate a confidence interval for the population estimate in this situation. In this approach, bootstrap samples are drawn from the primary observer counts and for each bootstrap sample, the population size is estimated by dividing the sum of the count by the proportion of the area surveyed. An assumption of this approach is that all or nearly all individuals in the surveyed units were observed. In our simulations, zero counts for the secondary observer were most common when the detection probability was high. Field data in which no birds are detected by either observer when acting as the secondary observer, but many birds are detected by primary observers, would indicate a high detection probability where the proposed bootstrap approach could be applied.

### Conclusions

For any study involving population size or density estimation, the choice of logistically feasible and cost-effective methods will be limited by the species, location, habitat, and study objectives. The DO and DS methods can be applied in many different settings and our evaluations provide important information on the performance of these two methods that can assist researchers in selecting the method most appropriate for their particular needs. Of the two estimation methods we evaluated, the DS method performed better in estimating the true population under a wide range of conditions. Population estimates from the DO method were highly variable when the detection probability was low and we agree with Nichols et al. (2000) that the DO method should not be used when the detection probability is less than 40%.

The two methods differed in confidence interval coverage, with coverage probabilities much closer to the nominal level with the DS method than the DO method. Where population estimation is a primary objective, the DS method may be preferable to the DO method if the assumptions of the method can be met, notably that the intensive method provide an unbiased estimate of the number of individuals present. However, if trend monitoring is the primary objective of the study, then either method could be used when the detection probability is expected to be high. In future work, it would be interesting to extend our investigation to include other population estimation methods, such as distance-sampling and N-mixture models.

## Materials and Methods

### Estimators

In the DS method, two survey techniques, a rapid and an intensive technique, are used to survey the study area. The study area is covered by *M* total survey units. From the *M* available survey units, *m_r_* are randomly selected to be surveyed with the rapid technique. Of the *m_r_* units surveyed with the rapid technique, *m*_i_ are randomly selected and surveyed with an intensive technique. The detection probability is estimated as 
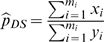
 where *x_i_* and *y_i_* are the number of birds recorded with the rapid and intensive techniques, respectively, in units surveyed with both techniques. Using the estimated detection probability and the counts obtained with the rapid technique covering the larger portion of the study area, the total population in the study area is estimated as 
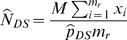
 with the variance estimated by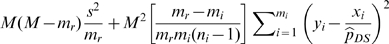 where 
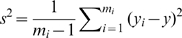
.

The derivation of these estimators is provided in Thompson [21]. For a sufficiently large sample, 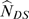 is approximately normally distributed and 95% confidence intervals can be constructed from the point estimate and estimated variance.

In the DO method, two observers survey the study area simultaneously. For each survey unit, the number of birds detected by observer *i* (*i* = 1, 2) when the alternate observer *j* (*j* = 1, 2) is the primary observer is designated as *x_ij_*. The counts for the secondary observer are of birds observed by the secondary observer but not detected by the primary observer whereas those for the primary observer consist of all detections by the primary observer only. Because the true number of birds in the surveyed area is not known, detection probabilities and population size are estimated by conditioning on the total number of birds detected by either observer (*x*_••_).

The maximum-likelihood estimator for the probability that a bird was observed by at least one observer is 
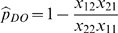
 [22]. Based on the total number of birds observed, the total population is estimated as 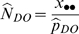 with its variance estimated by 
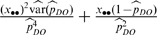
. A 95% confidence interval for 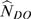 is 
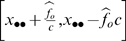
 where 
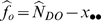
 and 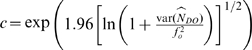. The derivation of these estimators is provided in [2], [22]. If only a portion of the study area is surveyed, then 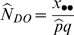 where *q* is the proportion of the study area surveyed. The variance is estimated as 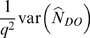. In this case, 
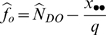
 and the 95% confidence interval becomes 
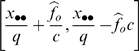
.

### Simulations

We first constructed three hypothetical populations of a single bird species in a study area consisting of 500 available survey units. Because birds are not evenly distributed throughout an area, but rather tend to be clustered, our three populations differed in the degree of population clustering. We randomly generated 500 values from one of three different gamma distributions, representing three levels of population clustering (Table 3). Shape and scale parameters for the gamma distributions were selected such that the expected value for the number of birds in each survey unit was 1.5. However, because each population was constructed by randomly generating 500 values, the total population size differed slightly among the three hypothetical populations. In the low cluster population (LC population), the number of birds in each survey unit varied from zero to five whereas in the high cluster population (HC population) many units had no birds, with a few containing relatively large numbers (10 or more). These values are comparable to counts per survey unit reported for real avian populations based on intensive survey methods (see e.g., [5], [15], [19]).

**Table 3:** Characteristics of simulated populations.

Clustering level	Shape, scale	Total population	SD (CV)
Low	3, 0.5	746	0.86 (0.58)
Medium	1, 1.5	752	1.65 (1.10)
High	0.25, 6	751	3.13 (2.1)

500 survey units were populated by drawing random variables from 1 of 3 gamma distributions with different shape and scale parameters selected to yield 3 levels of clustering and a mean of 1.5 birds per survey unit.

Next, we simulated 1,000 surveys of these populations and generated population estimates based on the DO and DS methods under a range of values for detection probability and proportion of the study area surveyed for each population. All simulations and analyses were conducted using R version 2.4 [23].

We evaluated detection probabilities ranging from 10% to 90%. For the DS method, the detection probability represented the probability of observing an individual with the rapid method. We assumed all birds were observed with the intensive survey technique of the DS method. For the DO method, the detection probability was the probability each observer had of detecting an individual present in a survey unit. In simulating the DO method, we initially used the same detection probability for each observer.

Because an assumption of the DO method is that all birds have the same probability of detection [2], we considered both constant and variable detection probabilities among individuals for both methods. To assess the effect of variable detection probabilities among individuals, we randomly generated a detection probability for each individual from a unimodal beta distribution with a mean equal to the target detection probability and standard deviation (SD) of 0.11 to 0.15.

We also investigated the effect of differences in detection probabilities between the two observers in the DO method. For this analysis, we considered all unique combinations of detection probabilities from 20% to 80% for each of the two observers and simulated 1,000 surveys of each population for each combination. Survey proportion was held constant at 50%.

We evaluated three levels for the proportion of the study area surveyed: 25%, 50% or 75%. For the DS method, the proportion surveyed represented the proportion surveyed with the rapid method. The proportion surveyed with the intensive method was initially set at 25% of the units surveyed with the rapid method, similar to proportions used in recent avian studies (31% [15], 19% [19] and 6% [5]). For the DS method, we also considered variations in the proportion of units surveyed with the intensive method. In this analysis, the proportion rapidly surveyed was held constant at 50% and the proportion of the rapidly surveyed units that were intensively surveyed set at 10%, 30%, and 50%.

For each population clustering level, detection probability and proportion surveyed, we generated data sets of observed survey counts for each method. Under some conditions (e.g., if the secondary observers see more unique birds than the primary observers for the DO method or for the DS method, if survey units selected for intensive survey contain no birds), population size and variance estimates cannot be calculated. For every combination of survey parameters investigated, we used 1,000 data sets for which both a population estimate and variance estimate could be calculated to compare the methods. From each simulated survey, we computed the estimated population size, bias and variance of the estimate, and 95% confidence intervals.

To simulate the DO method, the appropriate number of survey units was randomly selected from the 500 survey units according to the proportion of the study area to be surveyed. For each bird in a surveyed unit, a random number between zero and one was generated from a uniform distribution for each observer. If the random number generated for the primary observer was less than the bird's detection probability, the bird was seen by the primary observer. If the random number for the primary observer was greater than the detection probability, but the random number for the secondary observer was less than the detection probability, then the bird was seen by the secondary observer but not by the primary observer. This process was repeated for all birds in a survey unit to yield the number of birds seen by the primary observer and the number of birds seen by the secondary observer that were not seen by the primary observer for each survey unit.

To simulate the DS method, we randomly selected survey units for the rapid survey based on the proportion of the area to be surveyed. Units to be intensively surveyed were randomly chosen from those surveyed with the rapid method. For the rapid method, we determined the number of birds observed based on the detection probability and number of birds in the survey unit using the same procedure as for the DO method. For each bird in the survey unit, we randomly selected a number between zero and one. A bird was observed, if the random number was less than the bird's detection probability. We assumed all birds in a survey unit were observed with the intensive method.
